# Coronary artery calcification detected by initial polytrauma CT in severely injured patients: retrospective single-center cohort study

**DOI:** 10.1007/s00068-024-02487-x

**Published:** 2024-03-05

**Authors:** Hans-Jonas Meyer, Tihomir Dermendzhiev, Michael Hetz, Georg Osterhoff, Christian Kleber, Timm Denecke, Jeanette Henkelmann, Michael Metze, Robert Werdehausen, Gunther Hempel, Manuel F. Struck

**Affiliations:** 1 Department of Diagnostic and Interventional Radiology, University Hospital Leipzig, Liebigstr.20, 04103 Leipzig, Germany; 2 Department of Orthopedics, Trauma and Plastic Surgery, University Hospital Leipzig, Liebigstr. 20, 04103 Leipzig, Germany; 3 Department of Cardiology, Medical Department IV, University Hospital Leipzig, Liebigstr. 20, 04103 Leipzig, Germany; 4 Department of Anesthesiology and Intensive Care Medicine, University Hospital Leipzig, Liebigstr.20, 04103 Leipzig, Germany

**Keywords:** Trauma, Tracheal intubation, Coronary artery calcification, Weston score, CT

## Abstract

**Objectives:**

Coronary artery calcifications detected by computed tomography (CT) provide prognostic relevance for vascular disorders and coronary heart disease, whereas their prognostic relevance in severely injured trauma patients remains unclear.

**Material and Methods:**

All consecutive trauma patients requiring emergency tracheal intubation before initial CT at a level-1 trauma center and admission to the intensive care unit (ICU) over a 12-year period (2008–2019) were reanalyzed. The Weston score, a semiquantitative method to quantify coronary calcifications, was evaluated as a prognostic variable based upon whole-body trauma CT analysis.

**Results:**

Four hundred fifty-eight patients (74.6% male) with a median age of 49 years, median injury severity score of 26 points, 24-h mortality rate of 7.6%, and 30-day mortality rate of 22.1% met the inclusion criteria and were analyzed. Coronary artery calcification was present in 214 patients (46.7%). After adjustment for confounding factors, the Weston score was an independent predictor for 24-h mortality (hazard ratio, HR 1.19, 95% confidence interval, CI 1.06–1.32, *p* = .002) and 30-day mortality (HR 1.09, 95% CI 1.01–1.17, *p* = .027). In a subanalysis of 357 survivors, the Weston score was significantly associated with ICU length of stay (LOS) (beta weight 0.89, 95% CI 0.3–1.47, *p* = .003) but not with mechanical ventilation duration (beta weight 0.05, 95% CI -0.2–0.63, *p* = .304).

**Conclusion:**

CT-detected coronary calcification was a significant prognostic factor for 24-h- and 30-day-mortality in severely injured trauma patients requiring tracheal intubation, and influenced ICU LOS in survivors.

**Supplementary Information:**

The online version contains supplementary material available at 10.1007/s00068-024-02487-x.

## Introduction

Whole-body computed tomography (CT) is the established imaging modality for emergency diagnostics and is performed particularly in severely injured polytrauma patients. It is capable of providing all necessary trauma findings covering the whole body within minutes [1–3]. The findings comprise the detection of acute bleeding events, brain injuries, solid organ injuries, pneumothorax, spine injuries, pelvis fractures, and long bone fractures, which allows for the calculation of standardized scoring systems [4].

In addition to the diagnostic power of CT to detect injuries, the assessment of the prognostic relevance of other CT-derived injury markers and associated findings (e.g., body composition parameters or bone densitometry) has become increasingly popular [5, 6].

Coronary arterial calcium scoring is a biomarker to quantify the calcified plaque load of the coronary vessels, which are of prognostic importance in patients with coronary heart disease [7–10]. In general, coronary artery calcium is calculated on cardiac-gated CT images using the Agatston score [10], in contrast to the more recently developed Weston score, which is semiquantitatively calculated on nongated CT images and can therefore be calculated by the initial trauma CT [11–13].

There is a strong association between coronary artery calcium scoring and major cardiovascular events in asymptomatic individuals [10–14]. Moreover, it reflects the general vessel status, which could be of prognostic relevance in trauma patients, as comorbidities such as coronary heart disease and peripheral vessel disease might contribute to worse outcomes in older trauma patients [15, 16].

Published data on the use of coronary artery calcium scoring in trauma patients included relatively low injury severity profiles and subsequently low mortality rates, whereas studies including severely injured patients requiring tracheal intubation are currently not available [17, 18]. The need for tracheal intubation is an important clinical marker of injury severity, indicating potentially life-threatening conditions. It represents the gold standard to secure the airway and to maintain gas exchange in trauma patients with acute respiratory distress, hemodynamic shock, and impaired consciousness [19, 20].

The aim of the present study was to analyze the prognostic capability of coronary artery calcium measurements derived from initial trauma CT in a cohort of trauma patients who underwent emergency tracheal intubation. We hypothesized that coronary artery calcification, as a representative factor of the general cardiovascular condition, would be prognostically relevant in severely injured patients.

## Materials and methods

### Patient acquisition

After approval by the ethics committee at the Medical Faculty, Leipzig University, Leipzig, Germany (IRB00001750, project ID 441/15ek, September 14, 2020), consecutive trauma patients of the University Hospital Leipzig between January 2008 and December 2019 were retrospectively analyzed regarding the prognostic power of coronary artery calcium scoring adjusted for injury severity and demographic parameters, including preinjury condition. Informed consent was waived by the ethics committee since only anonymous data were analyzed and published and all data was obtained according to the human rights declaration of Helsinki. The inclusion criteria were direct admission from the scene to the emergency department (ED), presence of severe injuries requiring emergency tracheal intubation, performance of initial emergency CT diagnostics and admission to the intensive care unit (ICU). The exclusion criteria were patients younger than 18 years, incomplete or missing data, and CT imaging without contrast media use. Full compliance with the Strengthening the Reporting of Observational Studies in Epidemiology (STROBE) guidelines of cohort studies was provided (Table S1).

### Investigated parameters

Demographic parameters included sex, age, body mass index (BMI), and American Society of Anesthesiologists (ASA) classification. Injury severity was classified using the injury severity score (ISS), the presence of impaired consciousness was defined as Glasgow coma scale (GCS) ≤ 8 points, and the presence of shock at emergency department admission was defined as systolic blood pressure ≤ 90 mmHg. ICU length of stay (ICU LOS) in days, mechanical ventilation duration in days, and all-cause 24-h and 30-day mortality were assessed. All investigated parameters were obtained from paper-based and electronic patient charts and transformed into table format for further processing after anonymization of personal data (Table S2).

### Imaging technique

Contrast-enhanced CT was performed in a clinical setting using a 128-slice CT scanner (Ingenuity 128, Philips). Iodine-based contrast medium (90 mL Imeron 400 MCT, Bracco Imaging Germany GmbH) was administered intravenously at a rate of 2–4.0 mL/s. Automatic bolus tracking was performed in the descending aorta with a trigger of 100 Hounsfield units. CT images were obtained in the late arterial phase in every case. Typical imaging parameters were as follows: 100 kVp; 125 mAs; slice thickness, 1 mm; and pitch, 0.9. The CT covered the head to the upper thighs.

### Coronary artery calcification score – the Weston score

The assessment of the Weston score was performed by a single radiologist blinded to the clinical characteristics and outcome data (HJM). The four coronary arteries (left main (LCA), left anterior descending (LAD), right coronary (RCA), and left circumflex (RCX)) were scored for the extent of calcification on the CT images utilizing a 3-point scale. It ranges from 0 for no visible calcifications to 3 for extensive calcifications with blooming artifacts. The score is the sum of the four coronary arteries and therefore ranges from 0 to 12. It was initially described by Kirsch et al. and validated against cardiac-gated CT scoring [13].

### Statistical analysis

Data analysis included absolute numbers and proportions and medians and interquartile ranges (IQR, quartile 1 and quartile 3). After testing for normality distribution, group differences were calculated with the Mann‒Whitney U test, Student´s t test, and Chi-square test when appropriate. To identify independent predictors of 24-h and 30-day mortality, the Cox proportional hazard model was applied in which statistically significant predictors of univariable analyses were included in the multivariable model. In survivors, associations with ICU LOS and mechanical ventilation duration were analyzed using multivariable linear regression analyses, which included statistically significant predictors of univariable analyses. Hazard ratios, beta weights and 95% confidence intervals are provided. Correction for type 1 error was applied using the false discovery rate (FDR). In all instances, p values < 0.05 were considered statistically significant. The statistical analysis was performed using DATAtab (DATAtab e.U.) and GraphPad Prism version 10.0.2 for MacOS (GraphPad Software).

## Results

**Fig. 1:**
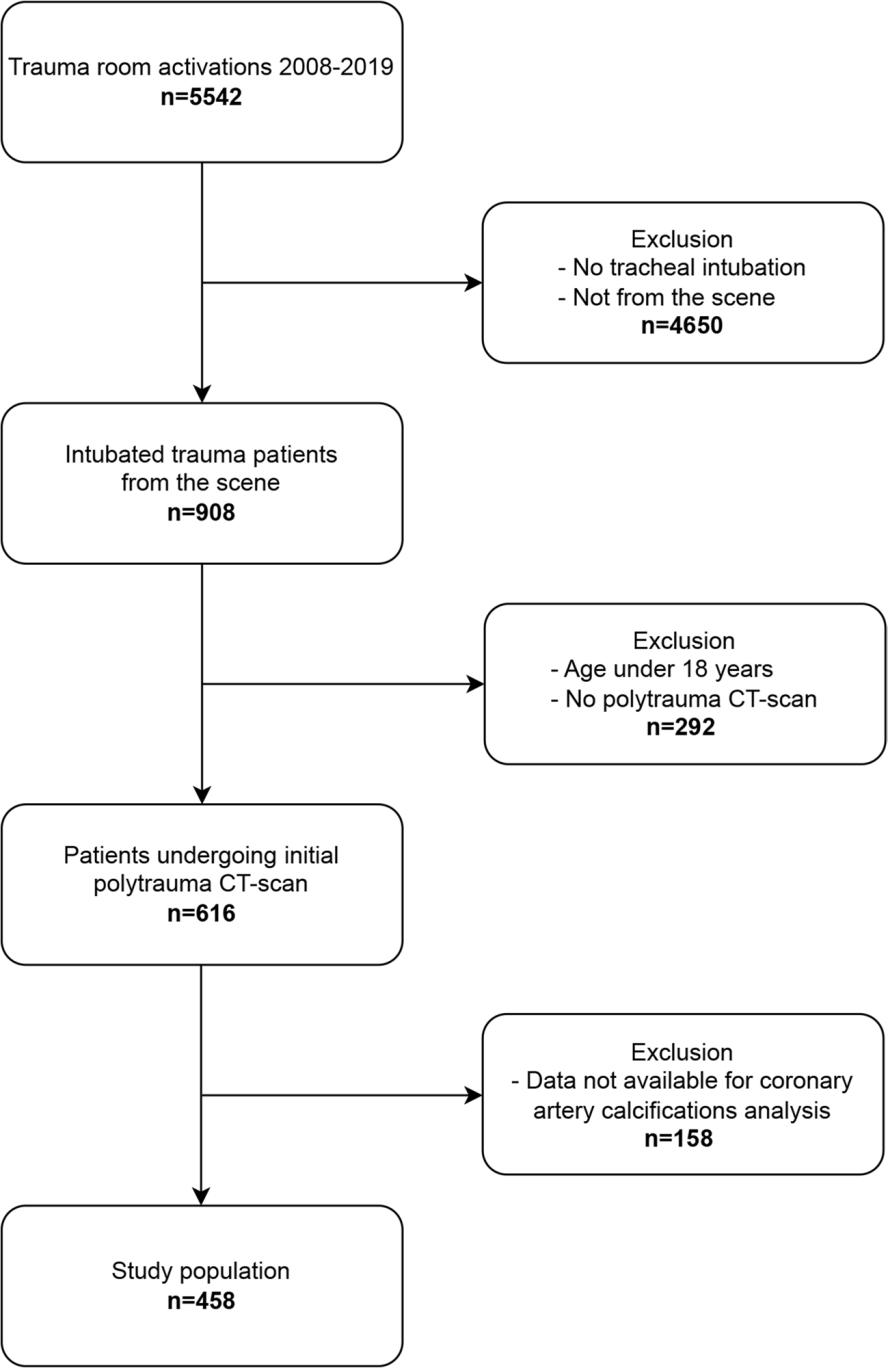
Study flow chart

Overall, 458 patients (342 male patients, 74.6%) with a median (IQR) age of 49 (31–64) years met the inclusion criteria (Fig. 1, Table S2). Traffic accidents were the main cause of injury (59%), whereas 31% of the patients had falls from height, 7% had other blunt injuries, and 3% had penetrating injuries. The median ISS was 26 (20–41) points, the median ICU LOS was eight (3–22) days, and the median mechanical ventilation duration was three (0.5–14) days. The all-cause 24-h mortality was 7.6% (35 patients) and 30-day mortality was 22.1% (101 patients) (Table 1).

**Table 1:** Baseline characteristics of patients with and without coronary artery calcification

Parameter	All patients (n = 458)	Weston score ≥ 1 (n = 214)	Weston score = 0 (n = 244)	p value
Male, n (%)	342 (74.7)	157 (73.3)	185 (75.8)	.547
Age, years; median (IQR)	49 (31–64)	61.5 (49.2–75)	35 (25–51)	< .001
BMI, kg/m^2^; median (IQR)	25 (23–28)	26 (24–28)	25 (23–27)	.009
ASA ≥ III; n (%)	100 (21.8)	85 (39.7)	15 (6.1)	< .001
ISS; median (IQR)	26 (20–41)	25 (20–41)	26.5 (20–38)	.935
Shock; n (%)	117 (25.5)	59 (27.57)	58 (23.8)	.352
GCS ≤ 8 points; n (%)	297 (64.8)	156 (72.9)	141 (57.8)	.001
ICU LOS, days; median (IQR)	8 (3–22)	10.5 (3–26)	7 (2–18.2)	.019
Mechanical ventilation, days; median (IQR)	3 (0.5–14)	4 (1–15)	3 (0.5–13)	.004
24-h mortality; n (%)	35 (7.6)	23 (10.8)	12 (4.9)	.019
30-day mortality; n (%)	101 (22.1)	58 (27.1)	43 (17.6)	.015

IQR, interquartile range; BMI, body mass index; ASA, American Society of Anesthesiologists classification; ISS, injury severity score; Shock, systolic blood pressure ≤ 90 mmHg at emergency department admission; GCS, Glasgow coma scale ≤ 8 points before tracheal intubation; ICU LOS, intensive care unit length of stay

### Weston score

**Fig. 2:**
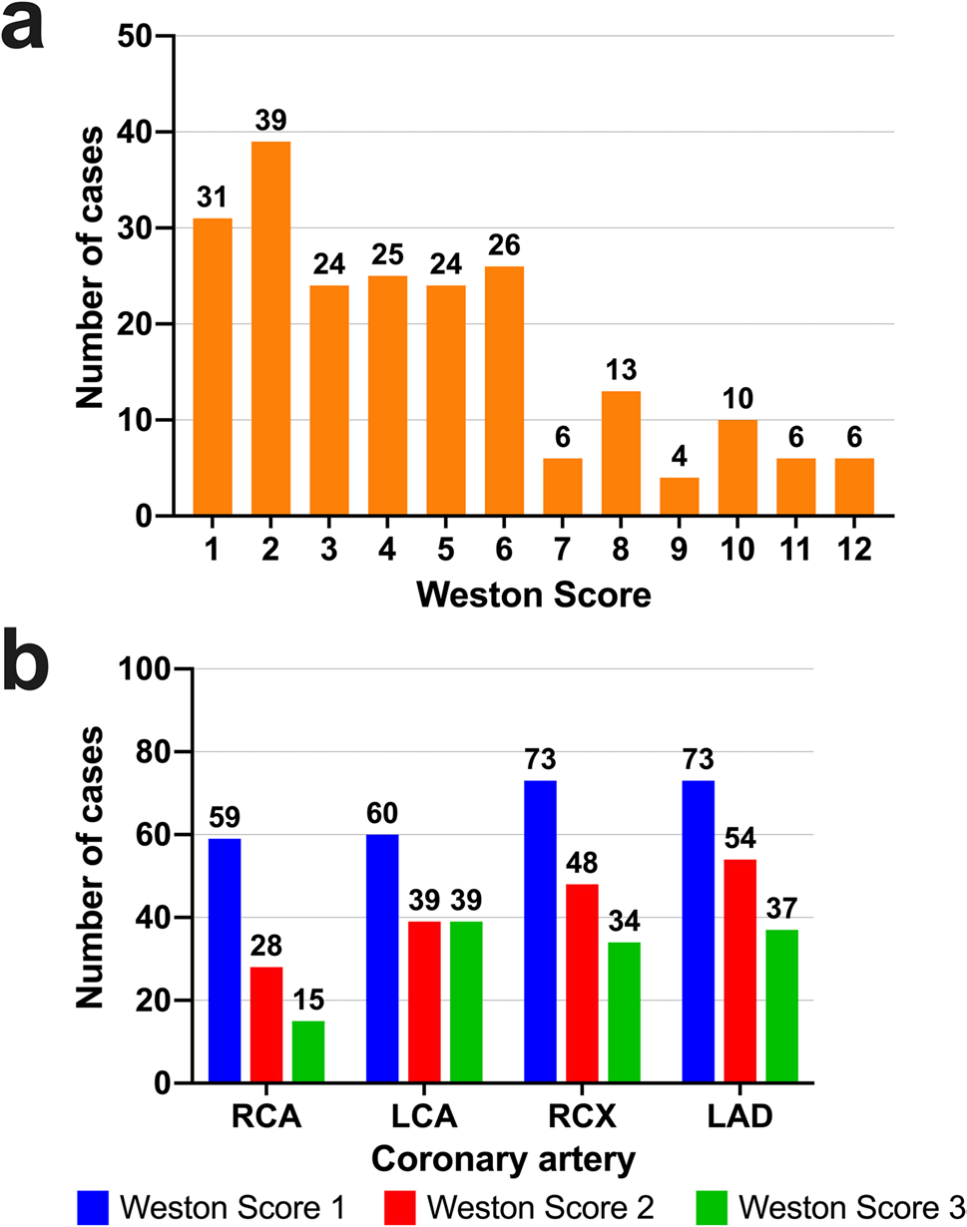
Frequency distribution of coronary artery calcification. The Weston score for the CT-detected degree of coronary artery calcification was assessed in 458 intubated trauma patients during their initial polytrauma CT scan with a maximum of three points for each of the four coronary artery sections. (**a**) Overall and (**b**) coronary artery section-specific Weston scores are displayed. RCA, right coronary artery; LCA, left coronary artery; RCX, ramus circumflexus (left circumflex artery); LAD, left anterior descending artery

**Fig. 3:**
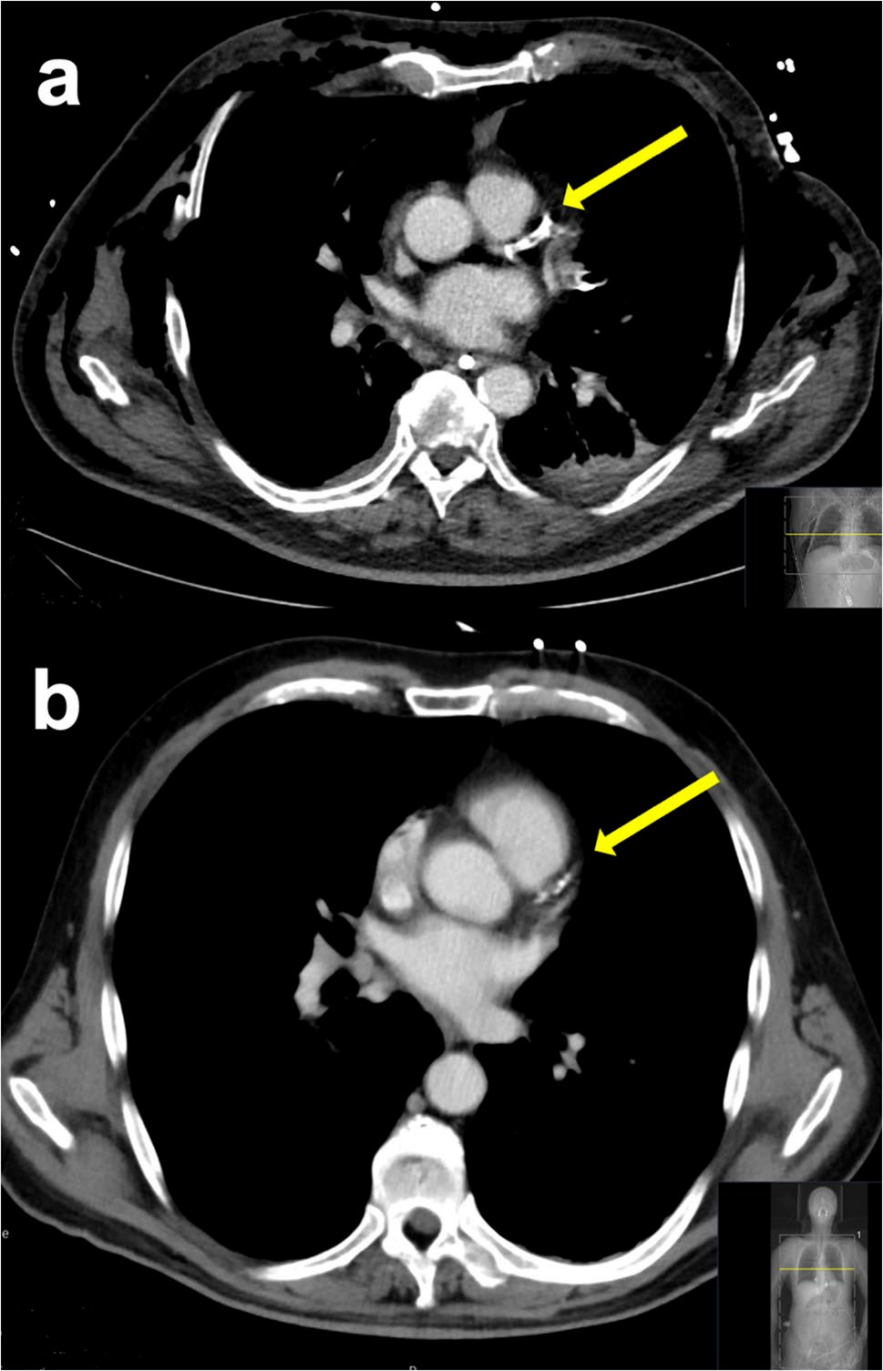
Two case examples of the left anterior descending artery (arrows) with extensive calcifications (**a**) and less calcifications (**b**) resulting in total Weston scores of 10 and 2 points, respectively

Overall, 214 patients (46.8%) had at least some visible calcification of the coronary vessels, whereas 244 patients (53.2%) had no visible calcifications on the CT images (Table 1). A total Weston score of up to 6 points was found in 80% of patients with coronary artery calcification. The exact proportions of coronary artery calcifications are shown in Fig. 2. Figure 3 provides two representative case examples from the present cohort.

Direct comparison of patients with and without coronary artery calcification revealed significantly higher median ages (61.5 years vs. 35 years, *p* < 0.001), higher median BMI (26 vs. 25, *p* = 0.009), higher proportions of ASA classification ≥ III (39.7% vs. 6.1%, *p* < 0.001), and higher proportions of GCS ≤ 8 points (72.9% vs. 57.8%, *p* = 0.001) in patients with coronary artery calcification (Table 1). Sex, ISS, and presence of shock were comparable in both groups (*p* > 0.05, each). Median ICU LOS (10.5 days vs. 7 days, *p* = 0.019), median mechanical ventilation duration (4 days vs. 3 days, *p* = 0.004), 24-h mortality rate (10.8% vs. 4.9%, *p* = 0.019) and 30-day mortality rate (27.1% vs. 17.7%, *p* = 0.015) were significantly higher in patients with coronary artery calcification.

### Associations with 24-h mortality

In the Cox proportional hazard model, statistically significant univariable associations with 24-h mortality were age, ASA classification ≥ III, Weston score, ISS, shock, and GCS ≤ 8 points (Table 2a). Sex and BMI were not significantly associated with 24-h mortality.

**Table 2:** Cox proportional hazard models of associations with 24-h and 30-day mortality

Parameter	Univariable HR	95% CI	p value	FDR	Multivariable HR	95% CI	p value	FDR
a) 24 h mortality								
Sex	0.84	0.4–1.74	.633	.704				
Age	1.02	1.01–1.04	.005	.007	0.99	0.96–1.01	.321	.321
BMI	0.98	0.9–1.08	.704	.704				
ASA ≥ III	4.27	2.2–8.31	< .001	< .001	3.92	1.53–10.03	.004	.008
Weston score	1.23	1.14–1.34	< .001	< .001	1.19	1.06–1.33	.002	.006
ISS	1.07	1.05–1.09	< .001	< .001	1.07	1.04–1.1	< .001	< .001
Shock	8.98	4.21–19.16	< .001	< .001	2.29	1–5.24	.049	.073
GCS ≤ 8 points	18.8	2.57–137.36	.004	.006	4.26	0.55–32.91	.165	.198
b) 30 day mortality								
Sex	0.92	0.59–1.43	.706	.706				
Age	1.02	1.01–1.03	.002	.003	1	0.99–1.02	.796	.796
BMI	1.05	1–1.11	.042	.048	1.03	0.98–1.1	.264	.308
ASA ≥ III	2.2	1.47–3.29	< .001	< .001	2.18	1.21–3.93	.009	.019
Weston score	1.12	1.06–1.19	< .001	< .001	1.09	1.01–1.17	.027	.037
ISS	1.06	1.05–1.07	< .001	< .001	1.05	1.04–1.07	< .001	< .001
Shock	4.79	3.21–7.21	< .001	< .001	2.16	1.37–3.41	.001	.003
GCS ≤ 8 points	5.38	2.61–11.09	< .001	< .001	2.72	1.26–5.84	.011	.019

HR, hazard ratio; CI, confidence interval; FDR, false discovery rate; BMI, body mass index; ASA, American Society of Anesthesiologists classification; ISS, injury severity score; Shock, systolic blood pressure ≤ 90 mmHg at emergency department admission; GCS, Glasgow coma scale before tracheal intubation

Adjusted for multiple variables, 24-h mortality was significantly associated with ASA classification ≥ III, Weston score, ISS, and shock (Table 2a). Age and GCS ≤ 8 points revealed no significant associations. After applying FDR adjustment, shock was nonsignificant.

Using only the three most significant associations for multivariable analysis to provide at least ten events per variable, statistical significance was confirmed in ISS (HR 1.09, 95% CI 1.07–1.11, *p* < 0.001), Weston score (HR 1.2, 95% CI 1.09–1.32, *p* < 0.001), and ASA classification ≥ III (HR 2.93, 96% CI 1.31–6.56, *p* = 0.009).

### Associations with 30-day mortality

Univariable analysis of 30-day mortality revealed statistically significant associations with age, BMI, ASA classification ≥ III, Weston score, ISS, shock, and GCS ≤ 8 points (Table 2b). Sex was not significantly associated with 30-day mortality.

Multivariable analysis confirmed ISS, shock, ASA classification ≥ III, Weston score, and GCS ≤ 8 points, as statistically significant associations, whereas age and BMI were nonsignificant.

### Associations with ICU LOS in survivors

In univariable linear regression analysis, age, ASA classification ≥ III, Weston score, ISS, shock, and GCS ≤ 8 points were significantly associated with ICU LOS (Table 3a). No significant associations were observed for sex and BMI. FDR adjustment resulted in nonsignificant association of ASA classification ≥ III.

**Table 3:** Linear regression analysis of associations with ICU LOS and mechanical ventilation duration in survivors

Parameter	B	Beta	SE	t	95% CI for B	p value	FDR
a) ICU LOS – univariable associations							
Sex	0.27	0.01	1.97	0.14	-3.62 to 4.16	.892	.892
Age	0.19	0.22	0.04	4.2	0.1 to 0.2	< .001	< .001
BMI	0.32	0.07	0.23	1.36	-0.14 to 0.78	.174	.198
ASA ≥ III	4.59	0.11	2.23	2.05	0.17 to 9	.041	.054
Weston score	1.19	0.2	0.31	3.89	0.59 to 1.8	< .001	< .001
ISS	0.66	0.51	0.06	11.27	0.54 to 0.77	< .001	< .001
Shock	11.17	0.25	2.26	4.39	6.69 to 15.65	< .001	< .001
GCS ≤ 8 points	9.85	0.3	1.64	6.01	6.61 to 13.1	< .001	< .001
b) ICU LOS – multivariable associations							
(Constant)	-12.03		2.55	-4.72	-17.07 to -6.99	< .001	
Age	0.16	0.19	0.04	3.83	0.08 to 0.24	< .001	< .001
Weston score	0.79	0.13	0.29	2.73	0.22 to 1.36	.007	.009
ISS	0.66	0.51	0.06	10.25	0.53 to 0.78	< .001	< .001
Shock	-0.14	0	2.12	-0.06	-4.33 to 4.06	.949	.949
GCS ≤ 8 points	4.05	0.12	1.46	2.78	1.17 to 6.92	.006	.009
c) Mechanical ventilation duration – univariable associations							
Sex	-1.74	-0.07	1.39	-1.25	-4.5 to 1.01	.211	.211
Age	0.14	0.24	0.03	4.61	0.08 to 0.21	< .001	< .001
BMI	0.35	0.11	0.16	2.11	0.02 to 0.67	.036	.041
ASA ≥ III	4.09	0.14	1.58	2.59	0.96 to 7.21	.01	.013
Weston score	0.61	0.15	0.22	2.78	0.18 to 1.05	.006	.009
ISS	0.43	0.47	0.04	10.02	0.34 to 0.51	< .001	< .001
Shock	4.6	0.15	1.64	2.8	1.35 to 7.85	.005	.009
GCS ≤ 8 points	9.4	0.41	1.11	8.45	7.2 to 11.6	< .001	< .001
d) Mechanical ventilation duration – multivariable associations							
(Constant)	-15.09		3.59	-4.21	-22.19 to -7.99	< .001	
Age	0.14	0.24	0.04	3.87	0.07 to 0.21	< .001	< .001
BMI	0.12	0.04	0.14	0.91	-0.15 to 0.4	.362	.422
ASA ≥ III	-0.99	-0.03	1.72	-0.57	-4.4 to 2.42	.566	566
Weston score	0.22	0.05	0.21	1.03	-0.2 to 0.63	.304	.422
ISS	0.44	0.48	0.05	9.59	0.35 to 0.53	< .001	< .001
Shock	-3.28	-0.1	1.51	-2.17	-6.26 to -0.3	.03	.053
GCS ≤ 8 points	5.81	0.25	1.03	5.64	3.78 to 7.85	< .001	< .001

ICU LOS, intensive care unit length of stay; B, unstandardized coefficient; Beta, standardized coefficient; SE, standard error; CI, confidence interval; FDR, false discovery rate; BMI, body mass index; ASA, American Society of Anesthesiologists classification; ISS, injury severity score; Shock, systolic blood pressure ≤ 90 mmHg at emergency department admission; GCS, Glasgow coma scale before tracheal intubation

Multivariable analysis confirmed age, Weston score, ISS, and GCS ≤ 8 points as independent associations of ICU LOS in survivors and did not confirm the association of shock (Table 3b).

### Associations with mechanical ventilation duration in survivors

In univariable linear regression analysis, age, BMI, ASA classification ≥ III, Weston score, ISS, shock, and GCS ≤ 8 points were significantly associated with the duration of mechanical ventilation, while sex was not significantly associated Table 3c).

Multivariable analysis confirmed age, ISS, shock, and GCS ≤ 8 points as independent associations of mechanical ventilation duration in survivors, whereas BMI, ASA classification ≥ III, and Weston score were not significantly associated (Table 3d). After FDR adjustment, the association of shock was not statistically significant.

## Discussion

Coronary artery calcification measured via Weston scoring was present in almost half of the study cohort of severely injured patients. The Weston score was independently associated with 24-h and 30-day mortality, suggesting a special vulnerability of patients in the acute trauma resuscitation setting. Furthermore, it was significantly associated with ICU LOS in survivors, whereas mechanical ventilation duration was similar in both groups. These results are a reminder of the importance of cardiac monitoring in severely injured trauma patients.

Patients with coronary arterial calcification had significantly higher ages, higher proportions of ASA classification ≥ III, and higher proportions of GCS ≤ 8 points than patients without coronary arterial calcification, which were the main confounders of the study cohort.

Only a few studies have investigated the prognostic relevance of coronary artery calcification scoring using initial trauma CT in severely injured patients [17, 18]. One study utilizing 592 patients aged 45 years or older with a median ISS of 13 points (IQR 6–25) and a mortality rate of 8.5% investigated different vessel calcifications of the aortic wall, coronary vessels and visceral vessels [17]. Notably, only calcification of the superior mesenteric artery was independently associated with mortality (Odds ratio (OR) 2.462, 95% CI 1.08–5.60, *p* = 0.032), whereas coronary artery calcification was not [17]. Moreover, the authors concluded that vascular calcifications were frequently observed (i.e., coronary arteries in 73% and abdominal arteries in 79.9% of the patients) [17]. Similar results were found in the aforementioned large prospective study cohorts investigating the prognostic role of coronary artery calcium scoring in asymptomatic individuals, underlining the importance of age adjustment to control for confounding factors [11–14].

Another study including 433 trauma patients with a median ISS of 9 points (IQR 5–14) and a mortality rate of 1.8% investigated coronary calcifications according to the number of calcified vessels with a score of 0–3 [18]. The authors reported an independent association between the number of calcified coronary vessels and the outcome of several complications, including infectious complications (OR 3.9, 95% CI 1.6–9.2) and delirium (OR 3.3, 95% CI 1.0–11.1) [18]. Furthermore, the number of calcified coronary arteries was a significant factor for an adverse discharge condition (*p* = 0.02). However, a comparison of these data with the present study cohort is not feasible due to considerably different injury severity and mortality rates.

Coronary artery calcification analysis is an underresearched field in trauma research, whereas it has extensively been evaluated as a relevant imaging biomarker throughout cardiovascular medicine [7, 9, 13, 14].

Recent results suggested that coronary artery calcification scoring is associated with mortality, ICU admission, and the need for mechanical ventilation in critically ill patients [21, 22]. A similar prognostic effect has been shown in unselected emergency patients [23], whereas the prognostic information may be independent of known cardiovascular risk factors [24]. While age – as shown in this present cohort – predicts coronary arterial calcification score, age may not be the sole major risk factor [25]. Elevated low-density lipoprotein cholesterol may correlate with elevated coronary arterial calcification scores [26], but even in patients with severely elevated low-density lipoprotein cholesterol, absent or low coronary arterial calcification scores have been observed [27]. This underscores the ability of CT to identify manifest arteriosclerotic disease. Since only approximately half of the patients with elevated coronary arterial calcification scores are on statin drugs [23], this opens the opportunity for cardiologic evaluation and pharmacologic prevention after surviving the acute trauma resuscitation phase and might further modify long-term prognosis.

Cardiac complications within the acute trauma resuscitation phase may be frequently observed and are likely the result of multiple factors. In one cohort of 343 trauma patients with a mean ISS of 28 points and an in-hospital mortality rate of 26.1%, 42.2% of the patients presented with myocardial injury defined as elevated troponin T levels, which were significantly associated with in-hospital mortality (adjusted OR 2.27, 95% CI 1.16–4.45, *p* = 0.017) [28]. Another analysis from this study group, which included a similar cohort of patients, revealed that the duration of hypotension in the resuscitation room was significantly associated with the presence of myocardial injury (OR 1.29, 95% CI 1.16–1.44, *p* = 0.012) [29]. In the present analysis, patients with and without coronary artery calcification had similar rates of shock, whereas shock was a significant predictor for all mortality classes. Although blunt chest contusion may trigger cardiac complications, particularly in preexisting coronary artery calcification [30, 31], the generation of elevated troponin levels after major trauma is associated with complex ischemia‒reperfusion mechanisms and physiological stress [32, 33].

The clinical value of measuring coronary calcium is to sensitize the trauma team with regard to cardiac complications and to draw the attention towards frequent cardiac evaluation (repeated ECG and transthoracic echocardiography) and secondary cardiac prevention (e.g. statin use, betablocker initiation, and platelet inhibition). The dilemma of the need for anticoagulation in coronary heart disease and the simultaneous presence of severe injuries requiring bleeding control requires an individual case-by case decision. After surviving the acute trauma resuscitation phase, posttraumatic cardiac care in patients at risk (identified using the Weston score) may also include early involvement of cardiologists to provide interdisciplinary expertise.

One strength of the present analysis is that the presence of coronary artery calcification was identified as a prognostic factor of our cohort, providing details regarding the exact extensions of the calcifications. This can easily be obtained by the radiologist in clinical routine and should be included in the standard report of the trauma patients. Although the present findings indicate only a moderate prognostic power of the Weston score compared with stronger predictors ISS and age, the establishment of routine measurement of coronary calcium might contribute to increase safety.

The limitations of the present study are, first, its single-center retrospective nature with possible known inherent bias. However, to reduce possible bias, the imaging analysis was performed blinded to the clinical information. Second, only patients requiring tracheal intubation and who underwent whole-body CT were included in this analysis. The need for advanced airway management during trauma resuscitation is a simple and pragmatic marker of considerable injury severity. Patients without tracheal intubation and those who did not receive initial whole-body CT due to direct transfer from the ED to the operating room, undergoing only head and/or chest CT, or who died in the ED may have presented with other predictors. Although these exclusions led to a more homogenous cohort with high overall injury severity scores and high mortality rates compared with previous analyses, they also increased the selection bias. Third, due to the retrospective nature of the study, it remains unclear whether some of the patients already had known coronary heart disease before the accident. Particularly in elderly patients who required CPR at the scene or during ED admission and died shortly after admission, cardiac causes of the accident might have been possible in some cases. Fourth, although the Weston score is a semiquantitative imaging analysis, we cannot exclude investigator-related bias. However, in the reported studies, it was highly correlated with the current gold standard of cardiac-gated CTs, the Agatston score, with low interrater variability [13]. Ongoing research develops deep learning algorithms to obtain coronary calcification scores in an automated manner, which will provide prognostic factors in a precise and time-efficient way under emergency conditions [34, 35].

In conclusion, coronary artery calcifications quantified by the Weston score in initial whole-body CT of severely injured trauma patients were significantly associated with 24-h and 30-day mortality and were furthermore associated with ICU LOS in survivors. More studies with cohorts of a relevant injury severity and comparable mortality rates are required to confirm the present findings.

## Supplementary Information

Below is the link to the electronic supplementary material.

Supplementary file1 (DOC 82 KB)

Supplementary file2 (XLSX 92 KB)

## Data Availability

The dataset supporting the conclusions of this article is included within the article (Table S2).

## References

[1] Huber-Wagner S, Lefering R, Qvick LM, et al. Effect of whole-body CT during trauma resuscitation on survival: a retrospective, multicentre study. Lancet. 2009;373:1455–1361. 10.1016/S0140-6736(09)60232-4. 19321199 10.1016/S0140-6736(09)60232-4

[2] Caputo ND, Stahmer C, Lim G, Shah K. Whole-body computed tomographic scanning leads to better survival as opposed to selective scanning in trauma patients: a systematic review and meta-analysis. J Trauma Acute Care Surg. 2014;77:534–9. 10.1097/TA.0000000000000414. 25250591 10.1097/TA.0000000000000414

[3] Chidambaram S, Goh EL, Khan MA. A meta-analysis of the efficacy of whole-body computed tomography imaging in the management of trauma and injury. Injury. 2017;48:1784–93. 10.1016/j.injury.2017.06.003. 28610777 10.1016/j.injury.2017.06.003

[4] Yoong S, Kothari R, Brooks A. Assessment of sensitivity of whole body CT for major trauma. Eur J Trauma Emerg Surg. 2019;45:489–92. 10.1007/s00068-018-0926-7. 29520416 10.1007/s00068-018-0926-7

[5] Sweet AAR, Kobes T, Houwert RM, et al. (2023) The association of radiologic body composition parameters with clinical outcomes in level-1 trauma patients. Eur J Trauma Emerg Surg 10.1007/s00068-023-02252-6 10.1007/s00068-023-02252-6 PMC10449658 36862245

[6] Kutleša Z, Ordulj I, Perić I, et al. Opportunistic measures of bone mineral density at multiple skeletal sites during whole-body CT in polytrauma patients. Osteoporos Int. 2023;34:775–82. 10.1007/s00198-023-06699-6. 36799980 10.1007/s00198-023-06699-6

[7] Greenland P, Blaha MJ, Budoff MJ, Erbel R, Watson KE. Coronary Calcium Score and Cardiovascular Risk. J Am Coll Cardiol. 2018;7:434–47. 10.1016/j.jacc.2018.05.027. 10.1016/j.jacc.2018.05.027 PMC6056023 30025580

[8] Jinnouchi H, Sato Y, Sakamoto A, et al. Calcium deposition within coronary atherosclerotic lesion: Implications for plaque stability. Atherosclerosis. 2020;306:85–95. 10.1016/j.atherosclerosis.2020.05.017. 32654790 10.1016/j.atherosclerosis.2020.05.017

[9] Nasir K, Cainzos-Achirica M. Role of coronary artery calcium score in the primary prevention of cardiovascular disease. BMJ. 2021;373:n776. 10.1136/bmj.n776. 33947652 10.1136/bmj.n776

[10] Agatston AS, Janowitz WR, Hildner FJ, et al. Quantification of coronary artery calcium using ultrafast computed tomography. J Am Coll Cardiol. 1990;15:827–32. 10.1016/0735-1097(90)90282-t. 2407762 10.1016/0735-1097(90)90282-t

[11] Budoff MJ, Nasir K, Kinney GL, et al. Coronary artery and thoracic calcium on noncontrast thoracic CT scans: comparison of ungated and gated examinations in patients from the COPD Gene cohort. J Cardiovasc Comput Tomogr. 2011;5:113–8. 10.1016/j.jcct.2010.11.002. 21167806 10.1016/j.jcct.2010.11.002 PMC3075464

[12] Shemesh J, Henschke CI, Shaham D, et al. Ordinal scoring of coronary artery calcifications on low-dose CT scans of the chest is predictive of death from cardiovascular disease. Radiology. 2010;257:541–8. 10.1148/radiol.10100383. 20829542 10.1148/radiol.10100383

[13] Kirsch J, Buitrago I, Mohammed TL, et al. Detection of coronary calcium during standard chest computed tomography correlates with multi-detector computed tomography coronary artery calcium score. Int J Cardiovasc Imaging. 2012;28:1249–56. 10.1007/s10554-011-9928-9. 21833776 10.1007/s10554-011-9928-9

[14] Ferencik M, Pencina KM, Liu T, et al. Coronary Artery Calcium Distribution Is an Independent Predictor of Incident Major Coronary Heart Disease Events: Results From the Framingham Heart Study. Circ Cardiovasc Imaging. 2017;10: e006592. 10.1161/CIRCIMAGING.117.006592. 28956774 10.1161/CIRCIMAGING.117.006592 PMC5659296

[15] Hashmi A, Ibrahim-Zada I, Rhee P, et al. Predictors of mortality in geriatric trauma patients: a systematic review and meta-analysis. J Trauma Acute Care Surg. 2014;76:894–901. 10.1097/TA.0b013e3182ab0763. 24553567 10.1097/TA.0b013e3182ab0763

[16] Sammy I, Lecky F, Sutton A, et al. Factors affecting mortality in older trauma patients-A systematic review and meta-analysis. Injury. 2016;47:1170–83. 10.1016/j.injury.2016.02.027. 27015751 10.1016/j.injury.2016.02.027

[17] De’Ath HD, Oakland K, Brohi K. CT screened arterial calcification as a risk factor for mortality after trauma. Scand J Trauma Resusc Emerg Med. 2016;24:120. 10.1186/s13049-016-0317-1. 27724913 10.1186/s13049-016-0317-1 PMC5057451

[18] Kobes T, Sweet AAR, Klip IT, et al. Cardiovascular parameters on computed tomography are independently associated with in-hospital complications and outcomes in level-1 trauma patients. Eur J Trauma Emerg Surg. 2023;49:1295–302. 10.1007/s00068-022-02168-7. 36436070 10.1007/s00068-022-02168-7 PMC10229702

[19] Crewdson K, Fragoso-Iniguez M, Lockey DJ. Requirement for urgent tracheal intubation after traumatic injury: a retrospective analysis of 11,010 patients in the Trauma Audit Research Network database. Anaesthesia. 2019;74:1158–64. 10.1111/anae.14692. 31069782 10.1111/anae.14692

[20] Anderson J, Ebeid A, Stallwood-Hall C. Pre-hospital tracheal intubation in severe traumatic brain injury: a systematic review and meta-analysis. Br J Anaesth. 2022;129:977–84. 10.1016/j.bja.2022.07.033. 36088135 10.1016/j.bja.2022.07.033

[21] Meyer HJ, Wienke A, Surov A. Extrapulmonary CT Findings Predict In-Hospital Mortality in COVID-19. A Systematic Review and Meta-Analysis. Acad Radiol. 2022;29:17–30. 10.1016/j.acra.2021.10.001. 34772618 10.1016/j.acra.2021.10.001 PMC8516661

[22] de Farias LD, Assuncao-Jr AN, Araújo-Filho JD, et al. 28-day prognostic value of coronary artery calcification burden in critically ill patients with COVID-19. J Cardiovasc Comput Tomogr. 2023;S1934–5925(23):00098–9. 10.1016/j.jcct.2023.03.012. 10.1016/j.jcct.2023.03.012 PMC10125832 37100677

[23] Chen L, Vavrenyuk A, Ren JH, et al. Prognostic Value of Coronary Artery Calcification Identified by the Semi-quantitative Weston Method in the Emergency Room or Other Hospitalized Patients. Front Cardiovasc Med. 2021;8:684292. 10.3389/fcvm.2021.684292. 34222379 10.3389/fcvm.2021.684292 PMC8248783

[24] Yu C, Ng ACC, Ridley L, et al. Incidentally identified coronary artery calcium on non-contrast CT scan of the chest predicts major adverse cardiac events among hospital inpatients. Open Heart. 2021;8:e001695. 10.1136/openhrt-2021-001695. 34635575 10.1136/openhrt-2021-001695 PMC8506889

[25] Bhatt SP, Kazerooni EA, Newell JD Jr, et al. Visual Estimate of Coronary Artery Calcium Predicts Cardiovascular Disease in COPD. Chest. 2018;154:579–87. 10.1016/j.chest.2018.05.037. 29890123 10.1016/j.chest.2018.05.037 PMC6130328

[26] Mortensen MB, Caínzos-Achirica M, Steffensen FH, et al. Association of Coronary Plaque With Low-Density Lipoprotein Cholesterol Levels and Rates of Cardiovascular Disease Events Among Symptomatic Adults. JAMA Netw Open. 2022;5:e2148139. 10.1001/jamanetworkopen.2021.48139. 35147685 10.1001/jamanetworkopen.2021.48139 PMC8837910

[27] Dong T, Tashtish N, Walker J, et al. Coronary Artery Calcium Scoring for Risk Assessment in Patients With Severe Hypercholesterolemia. Am J Cardiol. 2023;190:48–53. 10.1016/j.amjcard.2022.10.060. 36563458 10.1016/j.amjcard.2022.10.060

[28] Stroda A, Thelen S, M’Pembele R, et al. Incidence and prognosis of myocardial injury in patients with severe trauma. Eur J Trauma Emerg Surg. 2022;48:3073–9. 10.1007/s00068-021-01846-2. 34878581 10.1007/s00068-021-01846-2 PMC9360164

[29] Stroda A, Thelen S, M’Pembele R, et al. Association between hypotension and myocardial injury in patients with severe trauma. Eur J Trauma Emerg Surg. 2023;49:217–25. 10.1007/s00068-022-02051-5. 35920849 10.1007/s00068-022-02051-5 PMC9925499

[30] Kyriazidis IP, Jakob DA, Vargas JAH, et al. Accuracy of diagnostic tests in cardiac injury after blunt chest trauma: a systematic review and meta-analysis. World J Emerg Surg. 2023;18:36. 10.1186/s13017-023-00504-9. 37245048 10.1186/s13017-023-00504-9 PMC10225099

[31] Guo X, Wang X, Zhang X, et al. Acute myocardial infarction after blunt chest wall trauma with underlying coronary aneurysm: a case report. BMC Cardiovasc Disord. 2018;18:118. 10.1186/s12872-018-0861-x. 29914384 10.1186/s12872-018-0861-x PMC6006860

[32] Martin M, Mullenix P, Rhee P, et al. Troponin increases in the critically injured patient: mechanical trauma or physiologic stress? J Trauma. 2005;59:1086–91. 10.1097/01.ta.0000190249.19668.37. 16385284 10.1097/01.ta.0000190249.19668.37

[33] Edouard AR, Felten ML, Hebert JL, et al. Incidence and significance of cardiac troponin I release in severe trauma patients. Anesthesiology. 2004;101:1262–8. 10.1097/00000542-200412000-00004. 15564931 10.1097/00000542-200412000-00004

[34] Mu D, Bai J, Chen W, et al. Calcium Scoring at Coronary CT Angiography Using Deep Learning. Radiology. 2022;302:309–16. 10.1148/radiol.2021211483. 34812674 10.1148/radiol.2021211483

[35] Fan R, Shi X, Qian Y, et al. Optimized categorization algorithm of coronary artery calcification score on non-gated chest low-dose CT screening using iterative model reconstruction technique. Clin Imaging. 2018;52:287–91. 10.1016/j.clinimag.2018.08.015. 30193187 10.1016/j.clinimag.2018.08.015

